# Preoperative controlling nutritional status score (CONUT) predicts postoperative complications of patients with bronchiectasis after lung resections

**DOI:** 10.3389/fnut.2023.1000046

**Published:** 2023-01-20

**Authors:** Yong-sheng Cai, Xin-yang Li, Xin Ye, Xin Li, Yi-li Fu, Bin Hu, Hui Li, Jin-bai Miao

**Affiliations:** Department of Thoracic Surgery, Beijing Institute of Respiratory Medicine and Beijing Chao-Yang Hospital, Capital Medical University, Beijing, China

**Keywords:** bronchiectasis, operation, postoperative complications, nutritional status, CONUT scoring system

## Abstract

**Background:**

The Controlled Nutritional Status (CONUT) score is a valid scoring system for assessing nutritional status and has been shown to correlate with clinical outcomes in many surgical procedures; however, no studies have reported a correlation between postoperative complications of bronchiectasis and the preoperative CONUT score. This study aimed to evaluate the value of the CONUT score in predicting postoperative complications in patients with bronchiectasis.

**Methods:**

We retrospectively analyzed patients with localized bronchiectasis who underwent lung resection at our hospital between April 2012 and November 2021. The optimal nutritional scoring system was determined by receiver operating characteristic (ROC) curves and incorporated into multivariate logistic regression. Finally, independent risk factors for postoperative complications were determined by univariate and multivariate logistic regression analyses.

**Results:**

A total of 240 patients with bronchiectasis were included, including 101 males and 139 females, with an average age of 49.83 ± 13.23 years. Postoperative complications occurred in 59 patients (24.6%). The incidence of complications, postoperative hospital stay and drainage tube indwelling time were significantly higher in the high CONUT group than in the low CONUT group. After adjusting for sex, BMI, smoking history, lung function, extent of resection, intraoperative blood loss, surgical approach and operation time, multivariate analysis showed that the CONUT score remained an independent risk factor for postoperative complications after bronchiectasis.

**Conclusions:**

The preoperative CONUT score is an independent predictor of postoperative complications in patients with localized bronchiectasis.

## Introduction

Bronchiectasis is a chronic respiratory disease characterized by irreversible and permanent dilation of the bronchi, often accompanied by clinical symptoms such as hemoptysis and recurrent infections (1). Although many experts advocate conservative treatment to control symptoms, there is a high mortality rate of 19–31% (2). In addition, with the increasing incidence of bronchiectasis worldwide, lung resection plays an irreplaceable role in improving patient outcomes (3). However, it cannot be ignored that the reported complication rate after lung resection in patients with bronchiectasis is as high as 9.4–53% (4–8). Once postoperative complications occur, they not only increase the length of hospitalization and hospitalization costs but also seriously affect the prognosis of patients (9). Therefore, it is crucial to effectively identify the risk factors for postoperative complications in patients with bronchiectasis before surgery.

In recent years, the assessment of nutritional status has received increasing attention from surgeons. A large number of studies have shown that preoperative malnutrition is a high-risk factor for postoperative complications in many surgical procedures, such as gastrointestinal, hepatobiliary, and orthopedic procedures (10–14). Lee et al. demonstrated that preoperative nutritional status was an independent factor for postoperative complications in patients with non-small cell lung cancer who underwent pneumonectomy (15). Malnutrition and abnormal immune status are common in patients with bronchiectasis and are highly correlated with the severity of bronchiectasis and prognosis with conservative treatment (16–18). For example, Li et al. showed that serum albumin and prealbumin levels were highly consistent with the bronchiectasis severity index (16); Lee et al. demonstrated that body mass index (BMI) can be used as a predictor of bronchiectasis mortality (18). However, the role of preoperative nutritional status assessment in the surgical management of bronchiectasis and its association with postoperative complications are unclear.

Recently, several studies reported that preoperative nutrition scoring systems, including the Geriatric Nutrition Risk Index (GNRI), Prognostic Nutrition Index (PNI), and Glasgow Prognostic Score (GPS), could predict patient outcomes. Similar to these scoring systems, the Controlled Nutritional Status (CONUT) score, first proposed by Ignacio et al. (19), is a novel scoring system for evaluating preoperative nutritional status that includes serum albumin levels, peripheral blood lymphocyte counts and cholesterol levels, which reflect the nutritional status, lipid metabolism, and immune function of patients, respectively. By applying the COUNT score, clinicians can easily and comprehensively assess nutritional status. At present, extensive research supports the important role of the CONUT score in the prognosis of gastric cancer, esophageal cancer, lung cancer, hepatocellular carcinoma, and other surgical tumors (20–24). In recent years, CONUT scores have also shown good effects in predicting short-term postoperative complications (10–13). However, to our knowledge, no study has reported the association of the CONUT score with postoperative complications of bronchiectasis. This study aimed to investigate the clinical value of the preoperative CONUT score in predicting postoperative complications of bronchiectasis.

## Materials and methods

### Study population

We retrospectively analyzed clinical data from 240 patients with localized bronchiectasis who underwent lung resection at our hospital between April 2012 and November 2021. All patients were histopathologically confirmed. Inclusion criteria: (1) patients with localized bronchiectasis who received surgical treatment; (2) no nutritional supplementation during the perioperative period; and (3) complete clinical data. Exclusion criteria: (1) diffuse bronchiectasis or incomplete resection of bronchiectasis; (2) refusal of surgical treatment; (3) unable to tolerate surgery for any reason; (4) patients with malignant tumors. We collected patient information through the electronic medical record system as follows: basic information includes gender, age, body mass index (BMI), symptoms, duration of disease, preoperative comorbidities (tuberculosis, hypertension, diabetes, smoking, drinking, coronary heart disease), length of hospital stay (total length of hospital stay, length of hospital stay before operation, length of stay after operation); preoperative laboratory tests included: white blood cells, neutrophils, total lymphocytes, red blood cells, platelets, hemoglobin, albumin, total cholesterol; preoperative pulmonary function included forced expiratory volume in 1 s (FEV1) and the proportion of FEV1 to the predicted value; surgery-related data included surgical approach, extent of resection, operation time, intraoperative blood loss, drainage tube indwelling time and postoperative complications.

This study was approved by the Ethics Committee of Beijing Chaoyang Hospital Affiliated to Capital Medical University (2017-Ke-1). All procedures involving human participants in this study were performed in accordance with the ethical standards of the Institutional Research Council and the 1964 Declaration of Helsinki. Due to the retrospective design of the study, the requirement to obtain written informed consent from each patient was waived.

### Assessment of preoperative nutritional status

Blood samples were collected from all patients to complete the preoperative nutritional assessment. Patients undergoing emergency surgery had their blood drawn on the day of surgery for examination, and the rest of the patients had their blood drawn on the first day of hospitalization. The following three commonly used scoring systems were used to evaluate the nutritional status of patients before operation: control nutritional status score (CONUT), geriatric nutritional risk index (GRNI), and prognostic nutritional index (PNI). The CONUT score was calculated from the results of three laboratory tests, including serum albumin level, total lymphocyte count and cholesterol level (Supplementary Table). The calculation formula of the PNI score was as follows: 10^*^serum albumin level (g/dL) + 0.005^*^ total lymphocyte count (number/mm^3^) (25). The GNRI score was calculated as 14.89 ^*^ serum albumin level (g/dL) + 41.7 ^*^ (current weight/ideal weight), and the ideal weight was calculated as 22 ^*^ height squared (26). The optimal cutoff values for CONUT, GRNI, and PNI scores were determined according to the receiver operating characteristic (ROC) curve, which corresponded to the highest sensitivity and specificity. By comparing the area under the ROC curve (AUC), the best preoperative nutritional scoring system among the three was obtained.

### Surgical approach

Patients were selected for surgical treatment according to the following conditions: localized bronchiectasis confirmed by high-resolution computed tomography (HRCT); adequate cardiopulmonary reserve; clinical symptoms such as repeated coughing and expectoration, refractory massive hemoptysis and repeated pulmonary infections; and ineffective conservative treatment (27). Complete resection was defined as anatomic resection of all diseased segments on imaging.

All operations were performed by the same experienced surgeons. All patients were fully prepared before surgery until their condition was stable and then underwent surgery. The preoperative preparation included empirical antibiotic treatment and the use of atomization to improve the respiratory tract. All patients received general anesthesia by intravenous injection of propofol, and single-lung ventilation was established by double-lumen tracheal intubation. The surgeon decided to choose the surgical method (thoracotomy or video-assisted thoracoscopy) and extent of resection (pneumonectomy, lobectomy, lobectomy + lung segment/wedge and lung segment/wedge). If it is difficult to safely remove the lesion due to severe adhesion after entering the thoracic cavity, it can be converted to thoracotomy during the operation. Pulmonary vascular separation and dissection and lung tissue resection were performed using standard techniques. After careful hemostasis, all patients were covered with a new type of absorbable polyglycolic acid material, a Navi patch, and finally, a closed thoracic drainage tube was placed. The chest cavity was closed, and the operation was completed.

All specimens were histopathologically examined to confirm the presence of bronchiectasis. When the drainage volume of all patients was <200 ml/d after the operation, the color was clear, and there was no bubble overflow during coughing, the thoracic drainage tube was removed (28).

### Assessment of complications

Postoperative complications during hospitalization were the outcome variables of this study. According to the Clavien–Dindo classification system (29), the severity of postoperative complications in this study was Grade ≥ II. Postoperative complications were defined in patients with one of the following (30–32): prolonged air leak >7 days or more requiring intervention; pneumonia; hemorrhage; empyema; cardiac arrhythmias; bronchopleural fistula; wound infection; and atelectasis. Postoperative follow-up of the patients ended at discharge.

### Statistical analysis

All continuous variables were tested for normality. If they were not normally distributed, they were represented by M (Q1, Q3), and the Mann–Whitney *U*-test was used to analyze differences between groups. If they conformed to a normal distribution, they were expressed as the mean ± standard deviation, and the differences between groups were compared by Student's *t*-test. Categorical variables were expressed as numbers (%), using the χ2 test or Fisher's exact test. The optimal nutritional scoring system among the three was determined by receiver operating characteristic (ROC) curve and incorporated into the next step of multivariate logistic regression. Independent risk factors for postoperative complications in patients with bronchiectasis were identified by univariate and multivariate logistic regression. All statistical analyses were performed using IBM SPSS Statistics version 26.0 and R version 4.0.3 (version 4.0.3; http://www.Rproject.org), and a two-sided *P*-value of < 0.05 was considered as statistically significant.

## Results

### Patient characteristics

The characteristics of the patients in this study are shown in Table 1. A total of 240 patients were included, with an average age of 49.83 ± 13.23 years, including 101 males and 139 females, with an average body mass index of 23.13 ± 3.20. Hospitalizations due to recurrent infection, hemoptysis and other symptoms were 167 (69.6%), 145 (60.4%), and 15 (6.3%), respectively. The median duration of disease was 48 (5–120) months. A total of 169 (70.4%) patients underwent video-assisted thoracoscopic surgery (VATS), and 71 (29.6%) patients underwent thoracotomy. There were 15 (6.3%), 155 (64.6%), 55 (22.9%), and 15 (6.3%) patients who underwent pneumonectomy, lobectomy, lobectomy + segment/wedge resection and segment/wedge resection, respectively. The mean operation time was 153.10 ± 63.31 min, the median intraoperative blood loss was 100 (85–200) ml, the median total hospital stay was 11 (9–14) days, and the preoperative and postoperative median hospital stays were 5 (3–7) days and 6 (4–8) days, respectively.

**Table 1:** Baseline characteristics.

**Characteristic**	***N* = 240**
**Gender**, ***n*** **(%)**	
Male	101 (42.1)
Female	139 (57.9)
Age, years, mean ± SD	49.83 ± 13.23
BMI, kg/m^2^, mean ± SD	23.13 ± 3.20
**Symptoms**, ***n*** **(%)**	
Infection	167 (69.6)
Hemoptysis	145 (60.4)
Others	15 (6.3)
Duration of disease, months, median (range)	48 (5–120)
**Comorbidities**, ***n*** **(%)**	
Hypertension	43 (18.0)
Tuberculosis	29 (12.1)
Diabetes	21 (8.8)
Smoking	50 (20.8)
Drinking	28 (11.7)
CHD	7 (2.9)
**Preoperative laboratory tests, mean** ±**SD**	
WBC, × 109/L	6.62 ± 2.76
Neutrophils, × 109/L	4.18 ± 2.60
Total lymphocytes, × 109/L	1.87 ± 0.62
RBC, × 109/L	4.32 ± 0.60
Platelets, × 109/L	240.33 ± 63.97
Hb, g/L	129.17 ± 19.09
Albumin, g/L	40.24 ± 5.21
Cholesterol, mmol/L	4.11 ± 0.85
FEV1,L, mean ± SD	2.32 ± 0.74
**Surgical approach**, ***n*** **(%)**	
VATS	169 (70.4)
Open	71 (29.6)
**Extent of resection**, ***n*** **(%)**	
Pneumonectomy	15 (6.3)
Lobe	155 (64.6)
Lobe + Seg/Wed	55 (22.9)
Seg/Wed	15 (6.3)
Operation time, min, mean ± SD	153.10 ± 63.31
Blood loss, ml, median (range)	100 (85–200)
LOS, days, median (range)	11 (9–14)
LOS before surgery, days, median (range)	5 (3–7)
LOS after surgery, days, median (range)	6 (4–8)
Drainage duration, days, median (range)	4 (3–7)
Postoperative complication, *n* (%)	59 (24.6)

BMI, body mass index; CHD, coronary heart disease; WBC, white blood cells; RBC, red blood cells; Hb, hemoglobin; FEV1, forced expiratory volume in 1 s; VATS, video-assisted thoracoscopic surgery; Lobe, lobectomy; Seg/Wed, segmentectomy/wedge resection; LOS, length of hospital stay.

### Postoperative complications

A total of fifty-nine (24.6%) patients had sixty-seven complications (Table 2), and no patient died during hospitalization. Seventeen patients (7.1%) had persistent air leakage for >7 days, of which fourteen patients underwent lobectomy and three patients underwent lobectomy plus segment/wedge resection. Pneumonia occurred in twenty-eight patients (11.7%). Six patients (2.5%) had hemorrhage and underwent secondary surgery, including five patients with minimally invasive surgery and one patient with thoracotomy. Postoperative empyema occurred in two patients (0.8%), including one patient with minimally invasive pneumonectomy and one patient with minimally invasive lobectomy. Five patients (2.1%) developed arrhythmia. Two patients developed bronchopleural fistula (0.8%). Wound infection occurred in three (1.3%) patients. Postoperative atelectasis occurred in four (1.7%) patients.

**Table 2:** Postoperative complications after surgery for 240 patients with bronchiectasis.

**Postoperative complications**	**Number of patients (%)**
Persistent air leak for >7days	17 (7.1)
Pneumonia	28 (11.7)
Hemorrhage	6 (2.5)
Empyema	2 (0.8)
Cardiac arrhythmias	5 (2.1)
Bronchopleural fistula	2 (0.8)
Wound infection	3 (1.3)
Atelectasis	4 (1.7)

### Comparison of three nutritional scoring systems

Patients were divided into two groups according to whether complications occurred. The preoperative nutritional status of patients was evaluated by the CONUT score, GRNI score, and PNI score. The results showed that preoperative nutritional status was significantly correlated with postoperative complications regardless of the nutritional scoring system used (*P* < 0.05) (Table 3). The preoperative CONUT score of patients with postoperative complications was significantly higher than that of patients without complications (2.92 ± 2.22 vs. 1.73 ± 1.55, *P* < 0.001). The preoperative GRNI score (100.38 ± 11.37 vs. 105.07 ± 10.08, *P* = 0.003) and PNI score (38.78 ± 5.58 vs. 40.72 ± 5.01, *P* =0.012) of the patients with postoperative complications were significantly lower than those of the no complication group (Table 3).

**Table 3:** Relationship between CONUT score, GNRI score, PNI score and postoperative complications.

**Nutrition scoring system**	**Complications** **(*n* = 59)**	**No complications** **(181)**	***P***
CONUT	2.92 ± 2.22	1.73 ± 1.55	<0.001
GNRI	100.38 ± 11.37	105.07 ± 10.08	0.003
PNI	38.78 ± 5.58	40.72 ± 5.01	0.012

CONUT, controlling nutritional status; GNRI, geriatric nutrition risk index; PNI, prognostic nutritional index.

**Figure 1:**
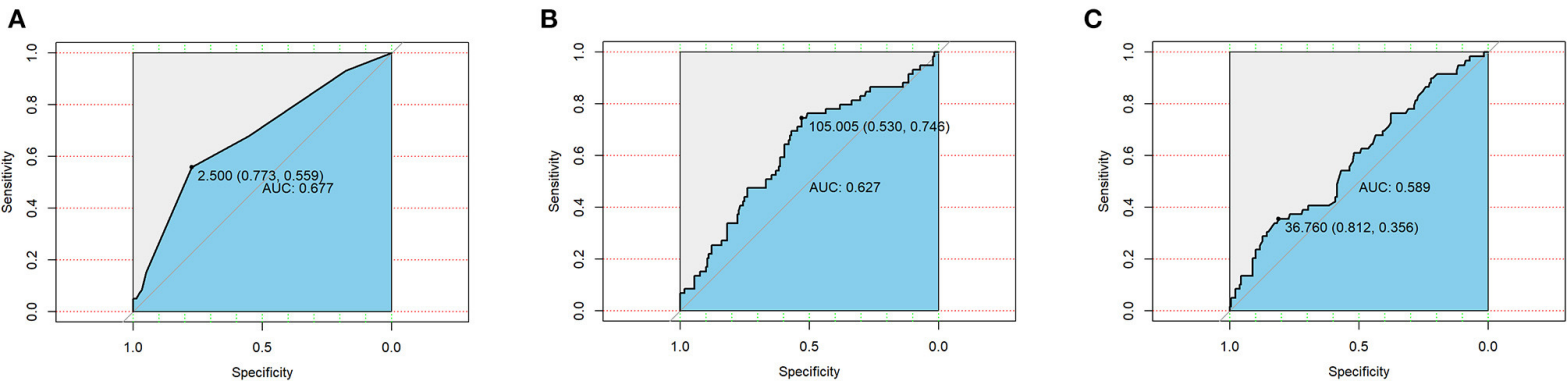
Receiver operating curves (ROC) of three nutritional scoring systems CONUT **(A)**, GNRI **(B)**, and PNI **(C)** in postoperative complications in patients with bronchiectasis.

Taking postoperative complications as outcome variables, ROC curves of the CONUT score, GRNI score, and PNI score were drawn, and the areas under the curve were 0.667, 0.627, and 0.589, respectively (Figure 1). This suggests that the CONUT scoring system is more suitable for assessing preoperative nutritional status in predicting postoperative complications in patients with bronchiectasis.

### Risk factors associated with postoperative complications

The risk factors for postoperative complications in patients with bronchiectasis were analyzed by univariate and multivariate logistic regression, and the results are shown in Table 4. Univariate analysis showed that sex, BMI, smoking history, CONUT score, and FEV1 were <60% of the predicted value, and the extent of resection, operation time, operation approach and intraoperative blood loss were significantly related to postoperative complications. Further multivariate logistic regression analysis showed that BMI (OR = 0.768, 95% CI: 0.614–0.962, *P* = 0.021), CONUT score (OR = 1.457, 95% CI: 1.023–2.076, *P* = 0.037), FEV1 <60% of the predicted value (OR = 8.220, 95% CI: 1.281–22.741, *P* = 0.026), and operation time (OR = 1.024, 95% CI: 1.008–1.041, *P* = 0.003) were independent risk factors for postoperative complications in patients with bronchiectasis.

**Table 4:** Univariate and multivariate analyses of risk factors associated with postoperative complications.

**Characteristics**	**Univariate**		**Multivariate**	

	**OR** **(95% CI)**	***P***	**OR** **(95% CI)**	***P***
**Gender**				
Male	Ref		Ref	
Female	0.474 (0.261–0.860)	**0.014**	0.463 (0.072–2.966)	0.417
Age, years	1.012 (0.989–1.035)	0.322		
BMI, kg/m^2^	0.885 (0.803–0.976)	**0.014**	0.768 (0.614–0.962)	**0.021**
**Symptoms**				
Infection (no vs. yes)	1.117 (0.594–2.102)	0.731		
Hemoptysis (no vs. yes)	1.251 (0.680–2.303	0.471		
Duration of disease, months	1.000 (0.997–1.002)	0.738		
**Comorbidities**				
Hypertension (no vs. yes)	0.773 (0.347–1.723)	0.529		
Tuberculosis (no vs. yes)	1.740 (0.759–3.989)	0.191		
Diabetes (no vs. yes)	0.485 (0.138–1.709)	0.260		
Smoking (no vs. yes)	2.581 (1.326–5.026)	**0.005**	1.211 (0.130–11.317)	0.866
Drinking (no vs. yes)	1.848 (0.801–4.265)	0.150		
CHD (no vs. yes)	1.235 (0.233–6.540)	0.804		
**Preoperative laboratory tests**				
WBC, × 109/L	1.013 (0.912–1.126)	0.811		
Neutrophils, × 109/L	1.034 (0.927–1.153)	0.550		
Total lymphocytes, × 109/L	0.510 (0.307–0.807)	**0.009**		
RBC, × 109/L	0.656 (0.396–1.085)	0.101		
Platelets, × 109/L	0.997 (0.993–1.002)	0.290		
Hb,g/L	0.988 (0.973–1.004)	0.138		
Albumin, g/L	0.929 (0.877–0.985)	**0.014**		
Cholesterol, mmol/L	0.696 (0.484–1.002)	**0.044**		
CONUT score	1.368 (1.156–620)	**<0.001**	1.457 (1.023–2.076)	**0.037**
FEV1,L	0.683 (0.370–1.263)	0.224		
**FEV1/predicted, %**				
**≥60**	Ref			
**<60**	7.276 (1.931–17.420)	**0.003**	8.220 (1.281–22.741)	**0.026**
**Surgical approach**				
VATS	Ref			
Open	3.532 (1.904–6.552)	**<0.001**	1.847 (0.331–10.316)	0.485
**Extent of resection**				
Pneumonectomy	7.429 (1.226–45.005)	**0.029**	2.232 (0.060–82.927)	0.663
Lobe	1.896 (0.408–8.804)	0.414	1.054 (0.072–15.457)	0.970
Lobe + Seg/Wed	2.220 (0.445–11.077)	0.331	0.357 (0.016–8.032)	0.517
Seg/Wed	Ref		Ref	
Operation time, min	1.013 (1.007–1.018)	**<0.001**	1.024 (1.008–1.041)	**0.003**
Blood loss, ml	1.001 (1.000–1.002)	**0.002**	0.999 (0.998–1.000)	0.180

BMI, body mass index; CHD, coronary heart disease; WBC, white blood cells; RBC, red blood cells; Hb, hemoglobin; FEV1, forced expiratory volume in 1 s; VATS, video-assisted thoracoscopic surgery; Lobe, lobectomy; Seg/Wed, segmentectomy/wedge resection.

The bold values indicate the value of *p* < 0.05, which is statistically significant.

### Relationship of CONUT score with length of hospital stay and drainage tube indwelling time

The optimal cutoff value of the CONUT score determined by receiver operating characteristic (ROC) curve was 2.5, and the corresponding sensitivity and specificity were 0.559 and 0.773, respectively. The patients were divided into a high CONUT group and a low CONUT group according to the optimal cutoff value, and the differences in length of hospital stay and chest tube drainage time were compared. The results showed that the total length of hospital stay [13 (10–18.5) vs. 10 (8–13), *p* < 0.001], the length of hospital stay after operation [8 (5–10) vs. 5 (4–7), *p* = 0.001] and chest tube drainage time [6 (4–8.75) vs. 4 (3–6), *p* = 0.004] in the high CONUT group were significantly higher than those in the low CONUT group, but there was no significant difference in the length of hospital stay before operation between the two groups (Table 5).

**Table 5:** Relationship of CONUT score with length of hospital stay and drainage tube removal time.

**Variable**	**CONUT** **<2.5** **(*n* = 167)**	**CONUT** **> 2.5** **(*n* = 73)**	***P-*value**
LOS	10 (8–13)	13 (10–18.5)	<0.001
LOS before surgery	5 (3–6)	5 (3–8)	0.557
LOS after surgery	5 (4–7)	8 (5–10)	0.001
Drainage tube indwelling time	4 (3–6)	6 (4–8.75)	0.004

LOS, length of hospital stay.

The results of the study showed that postoperative complications significantly prolonged the length of hospital stay after the operation and chest tube drainage time. The results are shown in Table 6. The total length of hospital stay, length of hospital stay after operation and chest tube drainage time of patients with postoperative complications were significantly higher than those of patients without complications, but there was no significant difference in the length of hospital stay before operation.

**Table 6:** Subgroup analysis of the relationship between CONUT score, postoperative complications, length of hospital stay, and length of chest tube indwelling.

**Variable**	**CONUT <2.5**	**CONUT > 2.5**	***P-*value**
LOS (complications)	14 (11.75, 20.5)	16 (11, 21)	0.516
LOS (no complications)	10 (8, 11.75)	11 (9.5, 14)	**0.002**
*P*-value	**<0.001**	**<0.001**	
LOS before surgery (complications)	5 (3, 6.25)	6 (3.5, 7)	0.549
LOS before surgery (no complications)	5 (3, 6)	5 (3, 9)	0.761
*P*-value	**0.876**	**0.851**	
LOS after surgery (complications)	9.5 (7, 14.5)	11 (7, 15)	0.587
LOS after surgery (no complications)	5 (4, 6)	6 (4.5, 8)	**0.002**
*P*-value	**<0.001**	**<0.001**	
Drainage tube removal time (complications)	7 (5, 10.5)	7 (6, 10)	0.84
Drainage tube removal time (no complications)	4 (3, 5)	4.5 (3, 6.75)	**0.043**
*P*-value	**<0.001**	**<0.001**	

LOS, length of hospital stay.

The bold values indicate the value of *p* < 0.05, which is statistically significant.

Further analysis showed that in patients without postoperative complications, the total length of hospital stay in the high CONUT group [11 (9.5–14) vs. 10 (8–11.75), *p* = 0.002], length of hospital stay after operation [6 (4.5, 8) vs. 0.5 (4, 6), *p* = 0.002], and chest tube drainage time [4.5 (3–6.75) vs. 4 (3–5), *p* = 0.043] were significantly higher than those in the low CONUT group, but the two groups were significantly higher than those in the low CONUT group. There was no statistically significant difference in the length of hospital stay before the operation (Table 6).

## Discussion

In our study, we found that the preoperative CONUT score was an effective predictor of postoperative complications in patients with bronchiectasis. Among 240 patients with localized bronchiectasis, the incidence of complications after lung resection was 24.6%, the most common being pneumonia and air leakage, which is consistent with the results of previous studies and deserves the attention of thoracic surgeons (4–8).

Our study is the first to demonstrate a significant association between preoperative nutritional status and postoperative complications in patients with bronchiectasis. In previous studies, thoracotomy, operation time, intraoperative blood loss, and resection range were high-risk factors for complications after lung resection, which was also confirmed in this study. After adjusting for the above factors, preoperative nutritional status was still an independent risk factor for predicting postoperative complications in patients with bronchiectasis. Bronchiectasis is a typical chronic inflammatory disease that not only affects the patient's diet and leads to malnutrition but also causes systemic inflammatory depletion, which may lead to a decreased anabolic rate and increased catabolic rate (33). Therefore, the preoperative nutritional status of these patients undergoing surgical treatment should be fully evaluated to avoid major complications in the perioperative period. Several previous studies have investigated the association between nutritional parameters and bronchiectasis severity and mortality (16–18). Li et al. showed that serum albumin levels, prealbumin levels, and BMI were highly consistent with the Bronchiectasis Severity Index, with prealbumin levels showing the strongest correlation (16). A recent large-scale Korean study showed that low body mass index was associated with increased mortality in patients with bronchiectasis (18). Low-weight (BMI <18.5 kg/m^2^) patients had a 2.6-fold higher mortality rate than normal-weight patients (18.5 ≤ BMI ≥ 22.9 kg/m^2^). Thus, nutritional status directly affects the prognosis of patients with bronchiectasis. However, none of the above studies evaluated patients with bronchiectasis undergoing surgical treatment. Our study analyzed clinical data from 240 patients with bronchiectasis who had been surgically treated. We are the first to associate preoperative nutritional status with postoperative complications, and the results showed that preoperative nutritional status was an independent risk factor for postoperative complications in patients with bronchiectasis.

When evaluating the preoperative nutritional status of patients with bronchiectasis, although the three nutritional scoring systems, CONUT, GRNI, and PNI, are all related to postoperative complications, the CONUT score is slightly superior, possibly because the scoring system contains more comprehensive items. The CONUT score is calculated based on serum albumin concentration, total cholesterol concentration, and total lymphocyte count and is a comprehensive and objective novel preoperative nutritional scoring system. Serum albumin is often used to assess nutritional status and systemic inflammation, and studies have shown that patients with hypoalbuminemia tend to have more severe bronchiectasis (16, 17). In addition, Kabata et al. reported that preoperative albumin supplementation helped reduce the probability of postoperative complications (34), which may be due to hypoalbuminemia affecting tissue healing or immune response impairment (35). Total lymphocyte count is an important parameter reflecting immune status, and lower lymphocyte levels may be associated with an insufficient immune response, and it has been reported that patients with bronchiectasis often have abnormal lymphocytes (36). In addition, Matiello et al. demonstrated that cancer patients with low lymphocyte counts have a poorer prognosis (37). Finally, inflammation can lead to a decrease in total cholesterol levels, which in turn affects intracellular signaling and impairs the immune system, leading to poor tissue healing, partly reflecting the nutritional status of the patient (15, 38). Therefore, the comprehensive use of the above three indicators can more comprehensively reflect the preoperative nutritional status of patients with bronchiectasis.

The multivariate results of this study showed that BMI, CONUT score, FEV1 <60% of the predicted value, and operation time were independent risk factors for complications after bronchiectasis. There are few studies on postoperative complications in patients with bronchiectasis. Both previous studies reported that FEV1 <60% of the predicted value was an independent risk factor for postoperative complications (7, 27), which is consistent with our findings. In addition, these two studies also reported that a history of pulmonary tuberculosis and incomplete resection were independent risk factors for postoperative complications; however, these were not confirmed in our study. We considered this because only patients with limited bronchiectasis were included in our study, and all patients were completely resected. Second, there may be sample size or individual differences. In addition to the CONUT score, we considered BMI and operation time to be independent risk factors for postoperative complications in patients with bronchiectasis.

Our results showed that the length of hospital stay and chest tube drainage time were significantly higher in patients with postoperative complications than in patients without complications. Subsequent subgroup analysis showed that in the uncomplicated cohort, CONUT scores were significantly independently associated with hospital stay and chest tube drainage time, which was consistent with previous reports in the literature (39–42). Both studies by Ramos et al. and Jagoe et al. reported significantly longer postoperative chest tube drainage and hospital stays in malnourished lung cancer patients (39, 40). In addition, another study found that patients who were assessed as severely malnourished were hospitalized five times longer than those who were well-nourished (42). This may be due to decreased fibroblast proliferation, collagen synthesis, and neovascularization in malnourished patients, resulting in delayed wound healing and slower disease recovery, which in turn prolongs the length of hospital stay and chest tube drainage time (41). Therefore, we suggest that clinicians evaluate the nutritional status of patients with bronchiectasis preoperatively according to the CONUT score to reduce the length of postoperative hospital stay and chest tube drainage time.

There are some potential limitations of this study. First, this is a retrospective single-center study. Second, our study only considered postoperative complications during hospitalization and did not consider complications after discharge, which may underestimate the true incidence. Finally, the optimal cutoff value of the CONUT score for patients with bronchiectasis is still unclear, and more prospective, multicenter studies are still needed for further validation. We plan to continue to make useful explorations in the future.

## Conclusion

The preoperative CONUT score was an independent predictor of postoperative complications in patients with bronchiectasis. We suggest that clinicians screen out malnourished patients according to the CONUT score and provide appropriate nutritional support to reduce the incidence of postoperative complications.

## Data availability statement

The raw data supporting the conclusions of this article will be made available by the authors, without undue reservation.

## Ethics statement

The studies involving human participants were reviewed and approved by the Ethics Committee of Beijing Chaoyang Hospital Affiliated to Capital Medical University (2017-Ke-1). Written informed consent for participation was not required for this study in accordance with the national legislation and the institutional requirements.

## Author contributions

Y-sC and X-yL collected and analyzed the patient data and were the major contributors in writing this manuscript. XY, XL, and Y-lF participated in data analysis and methodological guidance. J-bM contributed to designing and critically revising the article. BH and HL contributed to the article review. All authors read and approved the final manuscript.
