# Supplementary material for: Preoperative controlling nutritional status score (CONUT) predicts postoperative complications of patients with bronchiectasis after lung resections
Source: Front Nutr. 2023 Jan 20;10:1000046. doi: 10.3389/fnut.2023.1000046 (PMC9895366; doi:10.3389/fnut.2023.1000046)
Supplement: Supplementary file 1 [file Table_1.DOCX]

| **Supplementary table Assessment of nutrition status by the CONUT Score** | | | | |
| --- | --- | --- | --- | --- |
| **Parameters** | Malnutrition status | | | |
|  | Normal | Light | Moderate | Severe |
| Serum albumin (g/dL) | ≥3.50 | 3.00–3.49 | 2.50–2.99 | <2.50 |
| Score | 0 | 2 | 4 | 6 |
| Total lymphocyte count | ≥1600 | 1200–1599 | 800–1199 | <800 |
| Score | 0 | 1 | 2 | 3 |
| Total cholesterol (mg/dL) | ≥180 | 140–179 | 100–139 | <100 |
| Score | 0 | 1 | 2 | 3 |
| Total score | 0–1 | 2–4 | 5–8 | 9–12 |

Abbreviation: CONUT,controlling nutritional status.
